# Whole genome sequence data of *Bacillus anthracis* strain 3B1 isolated from rice soil

**DOI:** 10.1016/j.dib.2025.111586

**Published:** 2025-04-29

**Authors:** Rosamond Chan, Kah-Ooi Chua, Kelly Wan-Ee Teo, Dedat Prismantoro, Nurul Shamsinah Mohd Suhaimi, Abdullah Bilal Ozturk, Nia Rossiana, Febri Doni

**Affiliations:** a Department of Biology, Faculty of Mathematics and Natural Sciences, Universitas Padjadjaran, Jatinangor 45363, West Java, Indonesia; b Centre for Research in Biotechnology for Agriculture (CEBAR), Universiti Malaya, Kuala Lumpur 50603, Malaysia; c Institute of Biological Sciences, Faculty of Science, Universiti Malaya, Kuala Lumpur 50603, Malaysia; d Department of Chemical Engineering, Faculty of Chemical and Metallurgical Engineering, Yildiz Technical University, Esenler, Istanbul 34220, Türkiye; e Department of Global Development, Cornell University, Ithaca, NY 14853, USA

**Keywords:** *Bacillus anthracis*, Genome annotation, MGI sequencing platform, Phylogeny, Rice, Whole genome sequencing

## Abstract

Strain 3B1 was isolated from the soil of rice field cultivated under the system of rice intensification (SRI) in Sukabumi, West Java, Indonesia. The genome of strain 3B1 was sequenced using the MGI DNBSEQ platform, followed by bioinformatics processing, including genome assembly and gene annotation using SPAdes and Prokka, respectively. The assembled genome had a total length of 5,137,985 bp, distributed across 70 contigs, with 5,364 genes identified. Strain 3B1 shared the highest 16S rRNA gene sequence identity including *Bacillus paranthracis, B. nitratireducens, B. cereus, B. paramycoides, B. tropicus*, and *B. anthracis*, in the range of 99.86 to 99.93%. Both 16S rRNA gene and core genes-based phylogenetic analyses placed strain 3B1 in the same clade with *B. anthracis* strain Ames within the *Bacillus* genus. The phylogenetic placement was supported by the highest average nucleotide identity (ANI) value of 98.1% and digital DNA-DNA hybridization (dDDH) value of 82.7% shared between the genomes of *B. anthracis* strain Ames and strain 3B1, indicating that 3B1 is a strain of *B. anthracis*. Further gene annotation revealed that the genome of strain 3B1 lacked the genes encoding for virulence factors such as the *pag, cya*, and *lef*. Nonetheless, this data provides valuable insights into the genomic feature of strain 3B1, which can be bioprospected for various biotechnological applications.

Specifications Table

**Table:** 

Subject	Microbiology
Specific subject area	Genomics
Type of data	Table, Figures
Data collection	*B. anthracis* strain 3B1 was isolated from the soil of rice field cultivated under the system of rice intensification (SRI) method. Genomic DNA was extracted from the pure culture of the bacterium. Sequencing was performed using the MGI DNBSEQ platform. After sequencing, quality control was conducted with FastQC, followed by trimming and size selection using Trimmomatic v0.39. The genome was *de novo* assembled using SPAdes v3.15.4. Gene annotation was carried out using Prokka v1.14.6. A 16S rRNA gene-based maximum-likelihood phylogenetic analysis was conducted using MEGA XI and core genes-based phylogenomic tree was performed using IQ-Tree. The species identity of strain 3B1 was corroborated by ANI and dDDH analyses.
Data source location	City/Town/Region: Nagrak Organik SRI Center (NOSC), Sukabumi, West Java Country: Indonesia Latitude longitude: 6°50′42.4"S 106°48′20.5"E
Data accessibility	Data is publicly available at the NCBI repository: BioProject accession: PRJNA1181961 BioSample accession: SAMN44575399 Genome accession: JBIYZP000000000 Assembly accession: ASM4594985v1 Direct URLs to data: https://www.ncbi.nlm.nih.gov/bioproject/PRJNA1181961 https://www.ncbi.nlm.nih.gov/biosample/SAMN44575399 https://www.ncbi.nlm.nih.gov/nuccore/JBIYZP000000000 https://www.ncbi.nlm.nih.gov/datasets/genome/GCA_045949855.1/ https://www.ncbi.nlm.nih.gov/Traces/wgs/JBIYZP01 Supplementary material available at https://doi.org/10.17632/8yymnvrgbg.1
Related research article	None

## 1 Value of the Data

• The data supports comparative genomic study of *B. anthracis* strain 3B1 isolated from the soil of rice field cultivated under SRI, enabling researchers to explore genetic variations and evolutionary relationships.
• The data offers valuable insights into the genomic features of *B. anthracis* strain 3B1, which can be bioprospected for various biotechnological applications.
• The data enables analysis of genetic diversity and genomic features between *B. anthracis* strain 3B1 isolates from rice field soil and those of the pathogenic strains.

## 2 Background

*Bacillus anthracis* is a spore-forming bacterium best known as the causative agent of anthrax, a disease affecting both humans and animals [1]. This bacterium is a non-motile, non-haemolytic, aerobic Gram-positive rod within the *B. cereus* group, which includes *B. cereus, B. thuringiensis, B. mycoides, B. pseudomycoides, B. weihenstephanensis, B. cytotoxicus,* and *B. toyonensis* [2]. While often associated with pathogenicity, strains of *B. anthracis* isolated from environmental sources, such as soil, may exhibit distinct characteristics compared to clinical isolates [3,4]. Strain 3B1, isolated from rice paddy soil, provides an opportunity to explore the genetic diversity and potential ecological adaptations of *B. anthracis* in non-host environments. Whole genome sequencing (WGS) of this strain offers valuable insights into its genetic composition, evolutionary history, and differentiation from other strains, broadening our understanding of *B. anthracis* in diverse settings.

## 3 Data Description

**Fig. 1:**
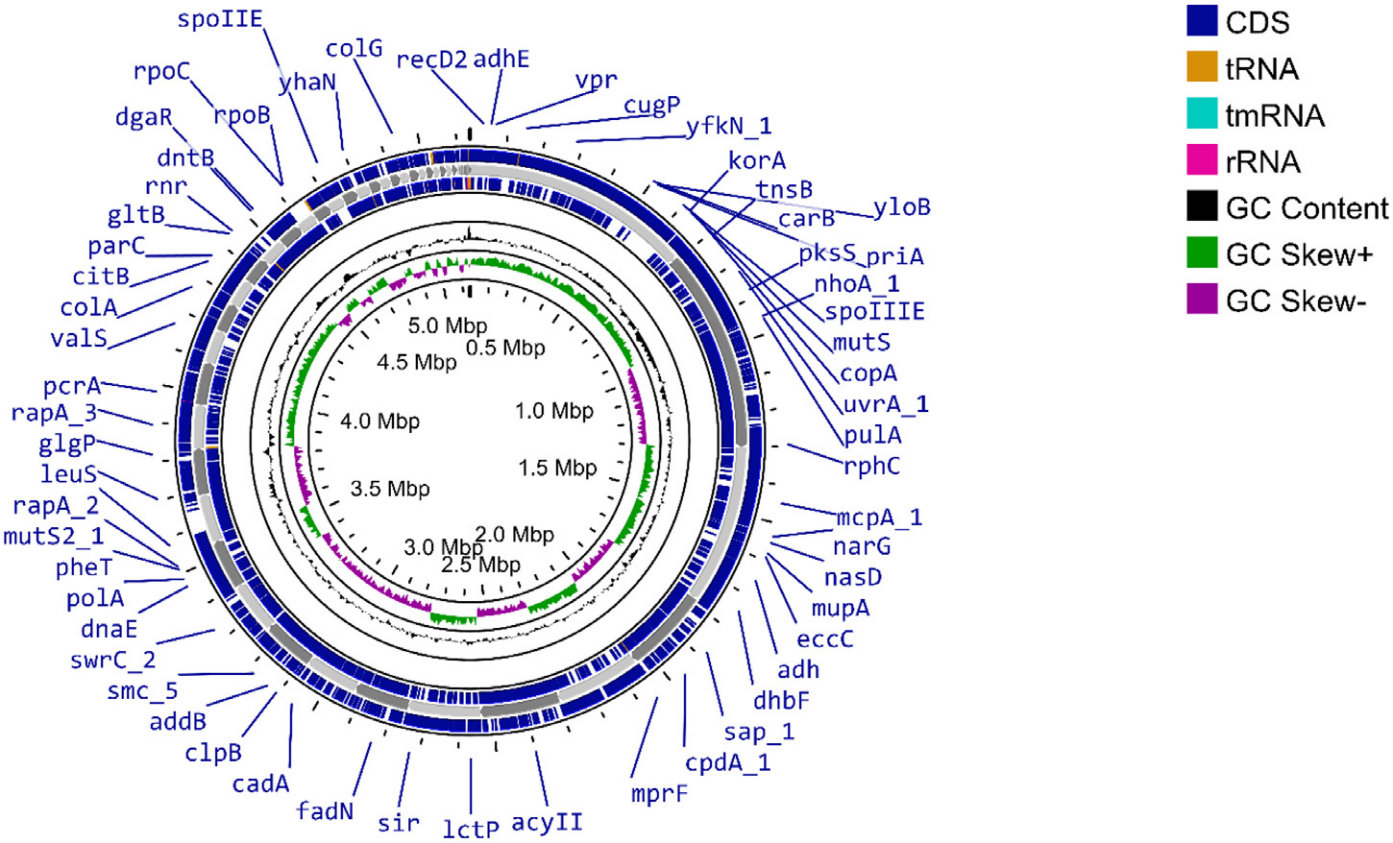
Visual representation of the strain 3B1 genome (∼5.13 Mbp) generated using the CGView Server (https://proksee.ca/).

We report the genomic data and analysis of strain 3B1, isolated from soil of rice field cultivated under SRI. The assembled genome is 5,137,985 bp in length, consists of 70 contigs, with an N_50_ contig length of 19,270 bp. The genome assembly exhibits a BUSCO completeness of 99.70% with minimal contamination and an average coverage of 72.95×. The genome has a GC content of 35.2%. Prokka annotation revealed 5,364 genes, including 5,201 coding DNA sequences (CDS), 66 tRNA genes, 6 rRNA genes, and 1 tmRNA gene (Table 1, Fig. 1).

**Table 1:** General genomic features of strain 3B1.

Features	Values
Genome size (bp)	5,137,985
G+C content (%)	35.2
Coverage	72.95×
No. of contigs	70
N_50_ (bp)	19,270
Genes (total)	5,364
CDS (coding sequences)	5,201
tRNA	66
rRNA	6
tmRNA	1

Strain 3B1 shares the highest 16S rRNA gene sequence similarities with several species within the genus *Bacillus*, including *Bacillus paranthracis, B. nitratireducens, B. cereus, B. paramycoides, B. tropicus*, and *B. anthracis*, in the range of 99.86 to 99.93%. A maximum-likelihood phylogenetic analysis of the 16S rRNA gene sequence placed *B. anthracis* strain 3B1 in the same clade with *B. anthracis* strain Ames but it was only supported by a low bootstrap value (Supplementary Fig. 1). These findings showed that strain 3B1 is a member of the *Bacillus* genus.

**Fig. 2:**
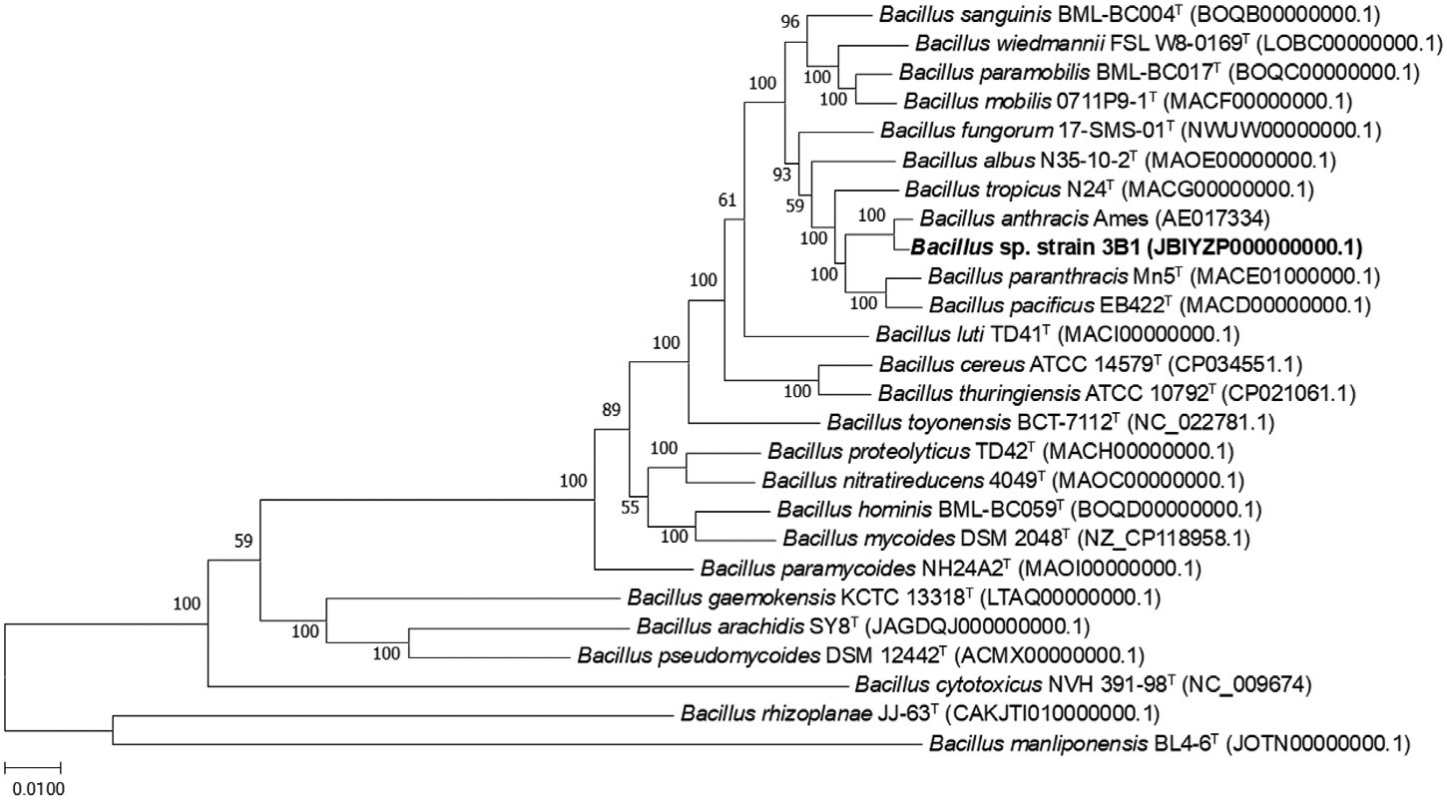
Phylogenomic tree based on core genes shared by strain 3B1 and closely related *Bacillus* species. Bootstrap values based on 1,000 replicates are indicated at the nodes.

Subsequently, the whole genome sequences of 25 *Bacillus* species available in the NCBI database were retrieved for a core genes-based phylogenomic analysis (Supplementary Table S1). Based on Roary analysis, these *Bacillus* strains shared a total of 112 core genes in their genomes. A maximum likelihood phylogenetic analysis based on all the core genes shared by the compared genomes placed strain 3B1 in the same clade with *B. anthracis* strain Ames. The phylogenetic placement based on analysis on the core genes was supported by a strong bootstrap value (100%) (Fig. 2), and proved a higher resolving power compared to 16S rRNA gene sequence alone. BTyper 3 analysis further confirmed that strain 3B1 is most closely related to *B. anthracis*, with a similarity value of 98.12%. In addition, strain 3B1 shared the highest average nucleotide identity (ANI) value at 98.1% (Supplementary Fig. 2) and the highest digital DNA-DNA hybridization (dDDH) value at 82.7% (Supplementary Fig. 3) with *B. anthracis* strain Ames. These findings corroborate that 3B1 is a strain of the species *B. anthracis*.

Prokka annotation and BTyper 3 results indicate that strain 3B1 lacks key anthrax virulence genes. These include *cya* (edema factor), *lef* (lethal factor), *pag* (protective antigen), and *atx* (anthrax toxin) [5], which are present in the reference *B. anthracis* strain Ames and play a crucial role in anthrax pathogenesis. Nonetheless, this data provides valuable insights into the genomic feature of strain 3B1, which can be bioprospected for various biotechnological applications.

## 4 Experimental Design, Materials and Methods

### 4.1 Isolation of strain 3B1

Strain 3B1 was isolated from soils collected from the rice field cultivated under SRI at the Nagrak Organik SRI Center (NOSC) in Sukabumi, West Java, Indonesia (6°50′42.4"S 106°48′20.5"E). The strain was grown in reinforced clostridial medium (RCM) broth and incubated at 30°C for 72 hours. The bacterial cells were then harvested and sent to Saraswanti Genomic Institute, Bogor, Indonesia, for whole genome sequencing.

### 4.2 Genomic DNA preparation

The DNA of strain 3B1 was extracted using the ZymoBIOMICS DNA Miniprep Kit (Zymo Research, Irvine, CA, USA) according to the manufacturer’s guidelines. The quality and concentration of the extracted DNA were evaluated using agarose gel electrophoresis and the Qubit BR-DNA Assay, respectively. Library preparation was performed using the MGIEasy FS DNA Library Prep Set (BGI, Shenzhen, Guangdong, China) according to the manufacturer’s protocol. Genome sequencing was conducted on the DNBSEQ-G400 (MGI Tech, Shenzhen, China) flow cell for paired-end sequencing, generating reads of 150 bp per end (PE150).

### 4.3 Whole genome sequencing

Whole genome sequencing (WGS) of strain 3B1 generated 4,036,996 raw reads. The quality of the raw sequence reads was evaluated using FastQC, followed by quality filtering with Trimmomatic v0.39 [6], which produced 2,997,550 high-quality reads with an average length of 150 bp. *De novo* genome assembly was conducted using SPAdes v3.15.4 [7], and the completeness of the assembly was assessed using BUSCO v5.7.1 [8,9]. A circular representation of the draft genome was generated using the CGView server (https://proksee.ca/) [10]. Gene prediction and annotation were performed using Prokka v1.14.6 [11].

### 4.4 Phylogenetic analyses

The 16S rRNA gene sequence was retrieved from the assembled genome and a BLAST search with reference to the EzBioCloud server (http://www.ezbiocloud.net/) was conducted to identify the closely related species [12]. The information about the reference species is shown in Supplementary Table 1. The 16S rRNA gene sequences of closely related type taxa were obtained from the NCBI database (http://www.ncbi.nlm.nih.gov/) and aligned in MEGA XI using ClustalW [13,14]. A 16S rRNA gene maximum-likelihood phylogenetic tree was constructed in MEGA XI with 1,000 bootstrap replicates. The whole genome sequences of closely related taxa were retrieved from the NCBI database. The core genes shared by all the compared genomes were identified using Roary (Galaxy v3.13.0) [15]. Core genes-based phylogenetic analysis was also conducted using IQ-Tree (http://iqtree.cibiv.univie.ac.at) [16] and the resulting tree was visualized using MEGA XI. Overall genome relatedness indices were calculated which include average nucleotide identity (ANI) analysis with fastANI [17,18] and BTyper 3 v3.4.0 [19], as well as digital DNA-DNA hybridization (dDDH) using the Genome-to-Genome Distance Calculator 3.0 server (https://ggdc.dsmz.de/ggdc.php) [20].

## Limitations

Not applicable.

## Ethics statement

This work does not involve human subjects or animal subjects. The authors declare that this manuscript is original work and has not been published elsewhere.

## Credit author statement

**Rosamond Chan**: Conceptualization, Software, Formal analysis, Investigation, Data curation, Visualization, Writing – review & editing, Writing – original draft; **Kah-Ooi Chua**: Software, Formal analysis, Data curation, Validation, Writing – review & editing; **Kelly Wan-Ee Teo**: Software, Formal analysis, Data curation; **Dedat Prismantoro**: Software, Formal analysis, Data curation; **Nurul Shamsinah Mohd Suhaimi**: Writing – review & editing; **Abdullah Bilal Ozturk**: Supervision; Writing – review & editing; **Nia Rossiana**: Supervision, Project administration, Funding acquisition, Writing – review & editing; **Febri Doni**: Project administration, Supervision, Funding acquisition, Writing – review & editing.

## Data Availability

NCBIBacillus anthracis strain 3B1, whole genome shotgun sequencing project (Original data)

## References

[1] Frey J., Prescott J.F., Rycroft A.N., Boyce J.D., MacInnes J.I., Van Immerseel F., Vázquez-Boland J.A. (2022). Pathogenesis of Bacterial Infections in Animals.

[2] Ehling-Schulz M., Lereclus D., Koehler T.M. (2019). The *Bacillus cereus* group: *Bacillus* species with pathogenic potential. Microbiol. Spectr..

[3] Banerjee A., Halder U., Chaudhry V., Varshney R.K., Mantri S., Bandopadhyay R. (2016). Draft genome sequence of the nonpathogenic, thermotolerant, and exopolysaccharide-producing *Bacillus anthracis* strain PFAB2 from Panifala hot water spring in West Bengal. India. Genome Announc..

[4] Salgado J.R.S., Rabinovitch L., Gomes M.F.S., Allil R.C.S., Werneck M.M., Rodrigues R.B., Picão R.C., Luiz F.B., de O., Vivoni A.M. (2020). Detection of *Bacillus anthracis* and *Bacillus anthracis*-like spores in soil from the state of Rio de Janeiro, Brazil. Mem. Inst. Oswaldo Cruz..

[5] Liang X., Zhu J., Zhao Z., Zheng F., Zhang H., Wei J., Ji Y., Ji Y. (2017). The *pag* gene of pXO1 is involved in capsule biosynthesis of *Bacillus anthracis* Pasteur II strain. Front. Cell Infect. Microbiol..

[6] Bolger A.M., Lohse M., Usadel B. (2014). Trimmomatic: a flexible trimmer for Illumina sequence data. Bioinformatics.

[7] Prjibelski A., Antipov D., Meleshko D., Lapidus A., Korobeynikov A. (2020). Using SPAdes De Novo assembler. Curr. Protoc. Bioinforma.

[8] Huang N., Li H. (2023). compleasm: a faster and more accurate reimplementation of BUSCO. Bioinformatics.

[9] Simão F.A., Waterhouse R.M., Ioannidis P., Kriventseva E.V., Zdobnov E.M. (2015). BUSCO: assessing genome assembly and annotation completeness with single-copy orthologs. Bioinformatics.

[10] Grant J.R., Enns E., Marinier E., Mandal A., Herman E.K., Chen C.-Y. (2023). Proksee: in-depth characterization and visualization of bacterial genomes. Nucleic. Acids. Res..

[11] Seemann T. (2014). Prokka: rapid prokaryotic genome annotation. Bioinformatics.

[12] Yoon S.H., Ha S.M., Kwon S., Lim J., Kim Y., Seo H., Chun J. (2017). Introducing EzBioCloud: a taxonomically united database of 16S rRNA gene sequences and whole-genome assemblies. Int. J. Syst. Evol. Microbiol..

[13] Tamura K., Stecher G., Kumar S. (2021). MEGA11: molecular evolutionary genetics analysis version 11. Mol. Biol. Evol..

[14] Thompson J.D., Gibson T.J., Higgins D.G. (2003).

[15] Page A.J., Cummins C.A., Hunt M., Wong V.K., Reuter S., Holden M.T.G., Fookes M., Falush D., Keane J.A., Parkhill J. (2015). Roary: rapid large-scale prokaryote pan genome analysis. Bioinformatics..

[16] Nguyen L.T., Schmidt H.A., Von Haeseler A., Minh B.Q. (2015). IQ-TREE: a fast and effective stochastic algorithm for estimating maximum-likelihood phylogenies. Mol. Biol. Evol..

[17] Musiał K., Petruńko L., Gmiter D. (2024). Simple approach to bacterial genomes comparison based on Average Nucleotide Identity (ANI) using fastANI and ANIclustermap. Folia Biol. Oecologica.

[18] Jain C., Rodriguez-R L.M., Phillippy A.M., Konstantinidis K.T., Aluru S. (2018). High throughput ANI analysis of 90K prokaryotic genomes reveals clear species boundaries. Nat. Commun..

[19] Carroll L.M., Cheng R.A., Kovac J. (2020). No assembly required: Using BTyper3 to assess the congruency of a proposed taxonomic framework for the *Bacillus cereus* group with historical typing methods. Front. Microbiol..

[20] Meier-Kolthoff J.P., Auch A.F., Klenk H.P., Göker M. (2013). Genome sequence-based species delimitation with confidence intervals and improved distance functions. BMC. Bioinformatics.

